# Association of Urinary Glyphosate with All-Cause Mortality and Cardiovascular Mortality among Adults in NHANES 2013–2018: Role of Alkaline Phosphatase

**DOI:** 10.3390/toxics12080559

**Published:** 2024-07-31

**Authors:** Yongyue Gao, Shuge Shu, Di Zhang, Pu Wang, Xiangyu Yu, Yucheng Wang, Yongquan Yu

**Affiliations:** 1 Second Affiliated Hospital of Guizhou University of Traditional Chinese Medicine, Guiyang 550000, China; gao188122@163.com (Y.G.); 122188@163.com (P.W.); 2 Key Laboratory of Environmental Medicine Engineering, Ministry of Education, School of Public Health, Southeast University, Nanjing 210000, China; ssg03300420@163.com (S.S.); yxy000923@163.com (X.Y.); 220213972@seu.edu.cn (Y.W.); 3 Key Laboratory of Modern Toxicology, Ministry of Education, School of Public Health, Nanjing Medical University, Nanjing 210000, China; zd211322@126.com

**Keywords:** glyphosate, cardiovascular disease, mortality, alkaline phosphatase, NHANES

## Abstract

Glyphosate is the most widely used herbicide in the world. This study aimed to evaluate the relationships among urinary glyphosate, all-cause mortality and cardiovascular diseases (CVD)-related mortality in the general US population of adults, and to determine the role of alkaline phosphatase (ALP), an inflammation marker that is associated with glyphosate exposure, in these relationships. Subjects from the National Health and Nutrition Examination Survey (NHANES) 2013–2018 cycles were included. Survey-weighted Cox regression analysis was applied to estimate the relationship of glyphosate with overall and CVD mortalities. Restricted cubic spline (RCS) analysis was utilized to detect the linearity of associations. The intermediary role of ALP was explored by mediation analysis. Our results found consistent and positive associations of glyphosate with all-cause mortality (HR: 1.29, 95%CI: 1.05–1.59) and CVD mortality (HR: 1.32, 95%CI: 1.02–1.70). RCS curves further validated linear and positive dose-dependent relationships between glyphosate and mortality-related outcomes. Moreover, serum ALP was identified as a mediator in these associations and explained 12.1% and 14.0% of the total associations between glyphosate and all-cause death and CVD death risk, respectively. Our study indicated that glyphosate was associated with increased all-cause and CVD mortality in humans. Increased ALP may play an essential role in these associations.

## 1. Introduction

Cardiovascular diseases (CVDs), a group of heart and vascular diseases including ischemic heart disease, cerebrovascular disease, rheumatic heart disease and congestive heart failure, are one of the foremost threats to human health [1]. The morbidity and mortality of CVDs have trended upward in recent years. It has been estimated that from 2010 to 2020, the prevalence of CVDs increased from 471.0 million to 607.6 million, equating to a 29.01% increase over approximately 10 years [2]. As the leading cause of death globally, CVDs were estimated to cause 18.6 million deaths in 2019, with an estimated increase to over 25.0 million by 2030 [3,4]. CVDs are also considered critical contributors to decreased quality of life and increased healthcare costs [5]. Therefore, identifying and controlling modifiable risk factors associated with CVDs have become top priorities. The etiology and development of CVDs appear to involve combined interactions between social, genetic and environmental factors [6,7], and among them, the contribution of environmental pollutants to CVDs has raised growing public concern. Several pollutants such as fine particulate matter, polycyclic aromatic hydrocarbons and heavy metals have been recognized as potential cardiovascular-toxic chemicals associated with increased CVD morbidity and mortality [8,9,10].

Glyphosate, a post-emergent and broad-spectrum herbicide, is ubiquitous throughout the environment [11]. Individuals are continuously exposed to glyphosate via means of drinking water, food ingestion, air inhalation and dermal contact [12]. Glyphosate is highly effective in inhibiting the shikimate pathway of the 5-enolpyruvylshikimate-3-phosphate synthase enzyme, which is mainly found in plants, fungi, bacteria, protozoa and archaea [13]. Thus, glyphosate exposure at environmental concentrations was believed to be “relatively safe” to mammals for a long time. However, recent epidemiological studies have revealed that urinary glyphosate exposure might be associated with increased risks of sex hormone disruption, osteoarthritis and cognitive disorders in humans [14,15,16]. Epidemiological studies have rarely explored the relationship between glyphosate and cardiovascular health; however, several animal-based studies have identified glyphosate as an emerging and widely distributed pollutant with cardiovascular-toxic potential [17,18]. For instance, environmental concentrations of glyphosate significantly inhibited cardiomyocyte proliferation and induced cardiovascular DNA damage, mitochondrial disruption and endoplasmic reticulum stress in zebrafish in a time- and dose-dependent manner [19]. In addition, in the offspring of glyphosate-treated pregnant rats, vascular function demonstrated decreased relaxation in response to acetylcholine and increased phenylephrine response in the aorta [20]. Therefore, the need for research focusing on the epidemiological association of glyphosate with cardiovascular health is growing in urgency.

Alkaline phosphatase (ALP) is a group of glycosylphosphatidylinositol-anchored glycoproteins that catalyze the dephosphorylation of phosphomonoester at alkaline pH [21]. Circulating ALP has been extensively applied as a predictor and risk marker of CVDs in routine clinical practice, and several population-based studies have revealed positive associations of serum ALP with CVDs and CVD-related mortality [22,23,24]. Mechanistically, ALP was linked to the increased calcification of vascular smooth muscle cells, activated systemic inflammation and disrupted cardiovascular oxidative balance [25,26,27]. In addition, a recent epidemiological study has demonstrated that glyphosate exposure was correlated with increased serum levels of ALP in the general US population [28], which was further supported by laboratory studies: glyphosate treatment significantly elevated circulating ALP levels and induced liver dysfunction in mouse [29], rat [30], and fish species [31]. Nonetheless, the potential role of ALP in glyphosate-related all-cause mortality and CVD mortality remains unclear.

Thus, in this study, epidemiological data were collected from the National Health and Nutrition Examination Survey (NHANES) from 2013 to 2018, in which urinary glyphosate concentration was measured. The associations of glyphosate with CVD risk and CVD cause-specific mortality were assessed using survey-weighted logistic regression and multivariate Cox regression, respectively. The mediating role of ALP in glyphosate-related cardiovascular injury was also evaluated using mediation analysis. By performing this study, we might provide novel clues and theoretical insights into the relationships among glyphosate, ALP and cardiovascular health.

## 2. Materials and Methods

### 2.1. Study Design and Participants

The NHANES refers to an ongoing, multistage designed and non-institutionalized health investigation that has been conducted to explore the health and nutritional status of individuals in the US since 1999. This survey is periodically performed by the National Center for Health Statistics (NCHS) in a 2-year cycle and contains publicly available data on the demographics, health behavior, disease status and environmental exposures of participants. The study protocol of NHANES was reviewed and approved by the Institutional Review Board (IRB) of the NCHS (Protocol #2011-17, #2018-01). Written informed consent was signed by all the participants before entering the survey, and none of the authors in the present study were involved in the production of NHANES.

**Figure 1:**
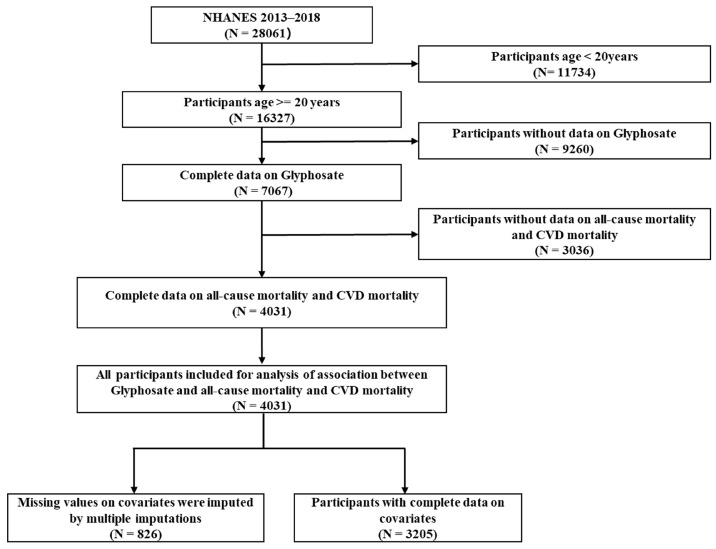
Flowchart of the study population screening in NHANES 2013–2018.

In our study, the study populations were drawn from three cycles of the NHANES (2013–2014, 2015–2016 and 2017–2018) in which glyphosate exposure data and CVD outcomes data were available simultaneously. Among the 28,061 subjects in the initial sample, those who were aged below 20 years old (N = 11,734) and had missing data on urinary glyphosate (N = 9260) were first excluded. Subjects with missing data on mortality outcomes were subsequently excluded (N = 3036). The ultimate study identified a total of 4031 subjects for all-cause mortality and CVD mortality analysis. The detailed screening process of subjects is depicted in the flow chart in Figure 1.

### 2.2. Measurements of Glyphosate Exposure

Spot urine specimens were collected at mobile examination centers, stored at a low temperature and transferred to the US Centers for Disease Control National Center for Environmental Health (CDC-NCEH). Two-dimensional on-line ion chromatography-tandem mass spectrometry (IC-MS/MS) coupled with isotope dilution quantification was used to detect the levels of urinary glyphosate by the Organic Analytical Toxicology Branch of CDC-NCEH. The concentration results were expressed as ng/mL, and the lower limit of detection (LLOD) of glyphosate was 0.2 ng/mL. The metabolites of glyphosate, such as aminomethylphosphonic acid (AMPA) and N-(phosphonomethyl)iminodiacetic acid, were not measured in the NHANES database. For more detailed experimental information, see the laboratory method part of the NHANES website (https://wwwn.cdc.gov/nchs/nhanes/Default.aspx (accessed on 10 March 2024)).

### 2.3. Ascertainment of CVD Outcomes

The NHANES database is linked to mortality data using death certificate records from the National Death Index. It is widely utilized as a prospective cohort with a large sample size and representative data including follow-up time, death status and leading cause of death. In this study, the information about death due to all-cause and CVDs was collected from the NHANES Linked Mortality File (LMF) until 31 December 2019. The International Classification of Diseases 10th Revision (ICD-10) codes were applied to determine the cause of mortality: death due to any causes from the ICD-10 codes was defined as all-cause mortality, while CVD cause-specific death was determined by ICD-10 codes I00–I09, I11, I13, I20–I51 or I60–I69. For more detailed mortality information related to the participants, see the NCHs website (https://wwwn.cdc.gov/nchs/nhanes/Default.aspx (accessed on 10 March 2024)).

### 2.4. Measurements of Serum ALP

Serum specimens were collected at mobile examination centers, stored at a low temperature and transferred to the CDC-NCEH. The levels of serum ALP were evaluated with the Beckman Coulter DxC800 Synchron clinical system (Beckman Coulter, CA, USA) using 2-Amino-2-Methyl-1-Propanol (AMP)-mediated kinetic methods. For more detailed experimental information, see the laboratory methodology part of the NHANES website (https://wwwn.cdc.gov/nchs/nhanes/Default.aspx (accessed on 10 March 2024)).

### 2.5. Covariates

Based on prior empirical literature [32], the following factors were identified as potential confounders and were controlled in this study: sociodemographic factors (sex, age, race/ethnicity, educational background, family poverty income ratio (PIR)), lifestyle factors (smoking status, drinking status and physical activity), hypertension, body mass index (BMI) and urinary creatinine. Among them, age, PIR and creatinine concentration were continuous variables, while the other covariates were categorical variables. Race/ethnicity was classified into “Other Hispanic”, “Non-Hispanic Black”, “Non-Hispanic White” and “Other Races” groups. Educational background was classified into “Less than high school”, “High school or equivalent” and “Some college or more” groups. BMI was classified into “<25 kg/m^2^”, “25–30 kg/m^2^” and “≥30 kg/m^2^” groups. Smoking status was determined based on the LLOD of cotinine (0.015 ng/mL), and subjects with serum cotinine levels ≥ LLOD were defined as smokers. Drinking status was determined based on the question “Had at least 12 alcohol drinks per year”, and subjects who answered “No” were defined as non-drinkers. In addition, alcohol consumers were further classified into “Moderate drinker (≤4 drinks/day)” and “Heavy drinker (>4 drinks/day)” groups based on the question “average alcoholic drinks per day”. The determinations of physical activity (“Vigorous”, “Moderate” and “Seldom”) and hypertension (“Yes” and “No”) were in accordance with our previous study [33].

**Figure 2:**
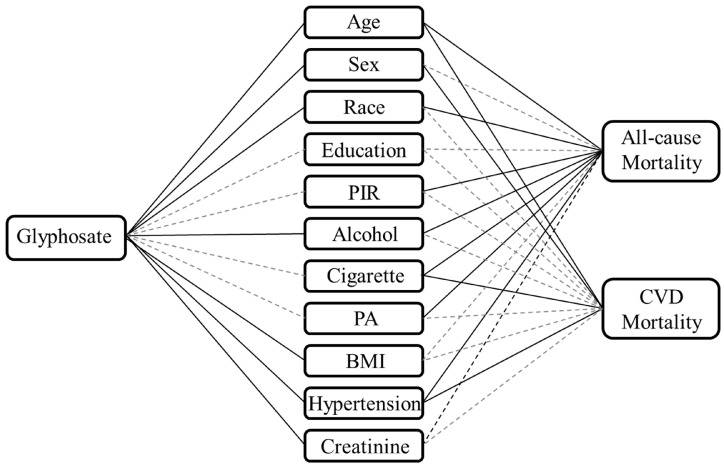
Confounder analysis of covariates with glyphosate exposure, all-cause mortality and CVD mortality. The solid lines indicate significant association between two variables, while the dotted lines indicate non-significant association. Abbreviations: CVD, cardiovascular diseases; PIR, family poverty income ratio; PA, physical activity; BMI, body mass index.

Confounder analysis was performed prior to incorporating the covariates into the analytic models. The potential correlations of covariates with glyphosate, all-cause mortality and CVD mortality are illustrated in Figure 2, and the education variable was excluded in the subsequent analyses.

### 2.6. Statistical Analysis

Continuous variables encompassing age, PIR, glyphosate, creatinine and ALP were displayed as the mean and standard deviation (SD), and comparisons between groups were performed using the Kruskal test or Student’s *t*-test. Categorical variables such as race, smoking status, drinking status and BMI were displayed as frequency and percentage (%), and comparisons between groups were performed using a chi-square test. Before regression analysis, the levels of urinary glyphosate and serum ALP were natural logarithm-transformed to reduce skewness.

Cox regression analysis was utilized to estimate the hazard ratios (HRs) of all-cause and cardiovascular mortality associated with glyphosate. The estimated coefficients were presented as HRs with 95% confidence intervals (CIs). As per the guidelines of NHANES, the complex design of NHANES and the subsample weights of urinary glyphosate were considered by including the variables for stratum, cluster and subsample weight. Three regression analysis models were introduced in sequence: model 1 was a crude model, model 2 was adjusted for lifestyle factors (including smoking status, drinking status and physical activity), hypertension and BMI, and model 3 was further adjusted for basic sociodemographic characteristics including age, sex, ethnicity and PIR based on model 2. Additionally, multivariate imputation by chained equation (MICE) was performed to calculate the missing values of the covariates (8.46% for PIR, 0.12% for educational background, 0.62% for BMI, 5.26% for drinking status, 2.78% for smoking status and 0.02% for urinary creatinine) to generate 10 datasets. Values for glyphosate concentration below LLOD (N = 974, 24.14%) were replaced by the constant value of LLOD/√2 as recommended by the NHANES. The estimates of Cox regression in each dataset were pooled to obtain the final result using Rubin’s rules. Several sensitivity analyses were conducted to test the robustness of our findings: (1) The associations of urinary glyphosate with all-cause mortality and cardiovascular mortality were reanalyzed without accounting for the complex sampling weights. (2) All the regression results were reanalyzed after excluding the subjects with missing data on covariates. 

To visualize the potential dose–response relationships of natural log-transformed glyphosate exposure with all-cause mortality and CVD mortality, and to test the nonlinearities of the above associations, restricted cubic splines (RCSs) with four knots (at the 5th, 35th, 65th and 95th centiles) were utilized in a fully adjusted model (the same covariates as model 3). Moreover, log-rank-based Kaplan–Meier survival curves were produced to display the univariate relationships between glyphosate and risks of all-cause mortality and cardiovascular mortality and to compare the survival times of glyphosate binary groups. 

Cox regression model-based mediation analysis was further conducted to evaluate the odds that exposure to glyphosate increased serum ALP levels and contributed to increased risks of all-cause mortality and CVD mortality. To test the robustness of mediation models, additional sensitivity analysis was performed by replacing glyphosate with BMI in the Cox regression model-based mediation analysis.

All the statistical analyses were analyzed using R software 4.1.2 (R Foundation for Statistical Computing, Vienna, Austria) except for Cox regression-based mediation analyses, which were conducted by STATA 15.0 (StataCorp, Lakeway Drive, College Station, TX, USA) using the macro “Med4way”. Two-sided *p*-value ≤ 0.05 was considered statistically significant.

## 3. Results

### 3.1. Baseline Characteristics of Selected Individuals

As shown in Table 1, the final study included 4031 eligible adults for mortality analysis. Overall, across the survey cycles, the average age and PIR of the selected individuals were 49.2 years and 2.554, respectively. The mean concentration of urinary glyphosate was 0.498 ng/mL, and the corresponding detectable rate was 75.86%. Most of the individuals were non-Hispanic White (N = 1613, 40.0%), lacked physical activity (N = 2262, 56.1%), were obese (N = 1627, 40.6%) and had high levels of cigarette exposure (N = 2627, 67.0%) and alcohol exposure (N = 2025, 53.0%). During the follow-up period, a total of 224 deaths, including 62 deaths caused by CVD, were observed. There were significant differences in urinary glyphosate level, all-cause mortality, CVD mortality, BMI, physical activity and smoking and drinking status between males and females (*p* < 0.05). However, no significant differences in other variables (age, race, PIR and hypertension) were identified.

### 3.2. Urinary Glyphosate Exposure, All-Cause Mortality and CVD Mortality

Table 2 reveals the results of survey-weighted univariate and multivariate Cox regression analyses of glyphosate with all-cause and CVD cause-specific mortalities. In the fully adjusted model (model 3), ln-transformed glyphosate was positively and significantly associated with both all-cause mortality and CVD mortality, and the corresponding HR was 1.29 (95%CI: 1.05 to 1.59) and 1.32 (95%CI: 1.02 to 1.70), respectively. The results of Cox regression analyses remained significant and positive in the univariate model (model 1, HR was 1.77 and 1.84 for all-cause mortality and CVD mortality, respectively) and the lifestyle-adjusted model (model 2, HR was 1.61 and 1.67 for all-cause mortality and CVD mortality, respectively). The majority of the results were stable in the sensitivity analyses (unweighted Cox regression analysis, survey-weighted Cox regression analysis with the missing value deleted and unweighted Cox regression analysis with the missing value deleted).

**Figure 3:**
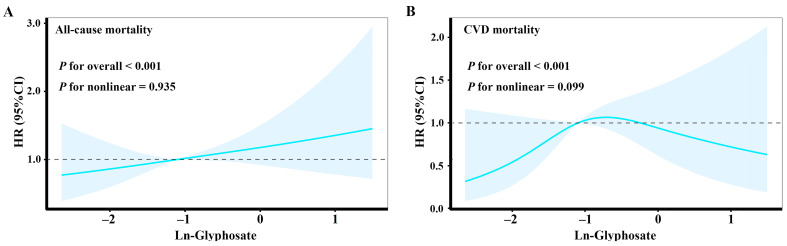
Association of glyphosate exposure with all-cause mortality and CVD mortality in restricted cubic spline curves. (**A**) Dose-response relationship between glyphosate and all-cause mortality in the general population of adults in the United States. (**B**) Dose-response relationship between glyphosate and CVD mortality in the general population of adults in the United States. The model was adjusted for sex, age, race/ethnicity, PIR, smoking status, drinking status, physical activity, hypertension, BMI and urinary creatinine. Abbreviations: CVD, cardiovascular diseases; PIR, family poverty income ratio; BMI, body mass index.

As shown in Figure 3, no significant nonlinearity was identified for the associations of glyphosate with all-cause and CVD mortality, with the *P* for nonlinearity being 0.935 and 0.099, respectively. The dose–response curves showed that the HR of all-cause mortality significantly increased with elevating glyphosate exposure. Although no apparent violation of linearity was observed, the RCS curve of CVD mortality shows an inverse U relationship with urinary glyphosate: the estimated HR of CVD mortality was first significantly increased and then slightly decreased as glyphosate concentrations increased.

**Figure 4:**
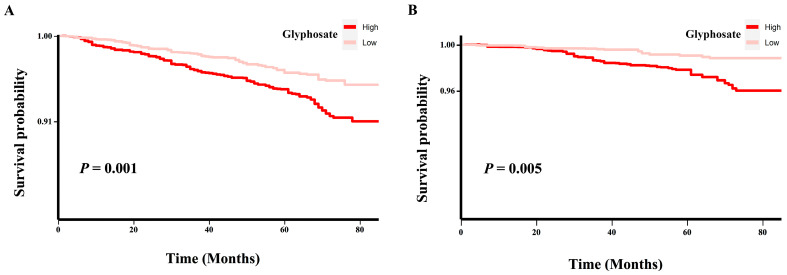
Kaplan–Meier survival curves of glyphosate exposure with all-cause mortality and CVD mortality. (**A**) Kaplan–Meier survival analysis of glyphosate and all-cause mortality in the general population of adults in the United States. (**B**) Kaplan–Meier survival analysis of glyphosate and CVD mortality in the general population of adults in the United States.

Furthermore, the curves of Kaplan–Meier analysis (Figure 4) showed that individuals in the high glyphosate exposure group had a higher survival rate from both all-cause and CVD death than those in the low glyphosate exposure group, with the corresponding *p* value being 0.001 and 0.005, respectively.

### 3.3. Role of ALP in the Associations of Glyphosate with All-Cause Mortality and CVD Mortality

**Figure 5:**
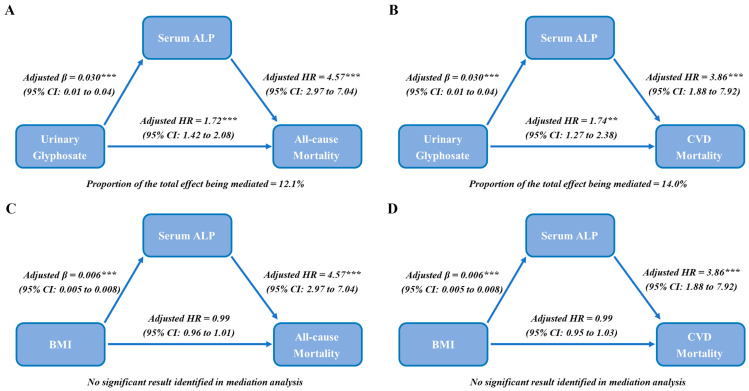
Role of ALP in the associations of glyphosate with all-cause mortality and CVD mortality. (**A**) Mediation analysis of ALP on the associations between glyphosate and all-cause mortality. (**B**) Mediation analysis of ALP on the associations between glyphosate and CVD mortality. (**C**) Mediation analysis of ALP on the associations between BMI and all-cause mortality. (**D**) Mediation analysis of ALP on the associations between BMI and CVD mortality. The model was adjusted for sex, age, race/ethnicity, PIR, smoking status, drinking status, physical activity, hypertension, BMI and urinary creatinine. Abbreviations: ALP, alkaline phosphatase; CVD, cardiovascular diseases; PIR, family poverty income ratio; BMI, body mass index. **: *p* < 0.01, ***: *p* < 0.001.

As shown in Figure 5, glyphosate was positively related to ALP in the fully adjusted model (β: 0.030, 95%CI: 0.010 to 0.040, *p* < 0.001). ALP was significantly associated with all-cause mortality and CVD mortality, with the corresponding HR being 4.57 (2.97 to 7.04) and 3.86 (1.88 to 7.92), respectively. The result of the mediation analysis showed that ALP significantly mediated the associations of glyphosate with all-cause mortality and CVD mortality, with the mediating ratios being 12.1% and 14.0%, respectively. Moreover, in sensitivity analysis, although BMI was significantly and positively associated with ALP (β: 0.006, 95%CI: 0.005 to 0.008, *p* < 0.001), no significant mediation analysis was identified in the associations among BMI, ALP, all-cause mortality and CVD mortality.

## 4. Discussion

In this study, by performing serial survey-weighted Cox regression and Kaplan–Meier analyses, we found significant associations between glyphosate exposure and increased all-cause mortality and CVD mortality in the general population of US residents (2013–2018). The results of RCS curves further confirm the contribution of glyphosate to mortalities and indicate that urinary glyphosate was linearly and dose-dependently correlated with all-cause mortality and CVD mortality. Meanwhile, glyphosate was positively associated with serum ALP levels, which were simultaneously linked to increased all-cause mortality and CVD mortality. Furthermore, ALP was recognized as a potential mediator that partially mediated the associations of glyphosate with mortality outcomes. To our knowledge, we are the first to detect the associations of urinary glyphosate with all-cause mortality and CVD mortality using a large sample size and to simultaneously consider the potential mediating effect of ALP.

As one of the highest selling and most frequently used herbicides in the world, glyphosate is ubiquitously distributed in household and industrial environments and has attracted increasing public attention [34]. Traces of glyphosate are frequently detected in human samples (including serum, urine, maternal milk, etc.) of both general and occupational populations [12]. As the absorbed glyphosate is mainly excreted in the urine, the levels of urinary glyphosate are often used to reflect recent glyphosate exposure [35]. Indeed, accumulating epidemiological studies have been performed to explore the associations between urinary glyphosate and adverse outcomes. For instance, in a prospective cohort study and a nested case-control study, the authors found that lifetime exposure to glyphosate (measured in urine samples) was associated with an increased risk of liver inflammation and metabolic syndrome in early adulthood [36]. The levels of glyphosate in first morning void urine were associated with increased oxidative stress biomarkers (8-hydroxy-2’-deoxyguanosine and malondialdehyde) in farmers that use glyphosate [37]. This study mainly focused on the cardiovascular problems relating to glyphosate in the general US population, as existing evidence on the relationship between glyphosate and CVD is rather limited. We also explored the mediating role of ALP in the associations between glyphosate and CVD mortalities, which might provide potential clues for the underlying mechanisms behind glyphosate-related CVD outcomes. As we found, urinary glyphosate was consistently and positively associated with all-cause and CVD mortalities, suggesting that the need for social measures to reduce human glyphosate exposure is growing more urgent.

Few studies have detected the cardiovascular toxicity of glyphosate in humans. Up to now, only one epidemiological study utilizing data from NHANES 2013–2016 has revealed positive associations of glyphosate exposure with several outcomes, including hypertension, obesity and CVD. However, the authors did not evaluate the association between glyphosate and CVD mortality; furthermore, they enrolled participants from a limited sample size [38]. In contrast, several experimental animal studies have demonstrated that glyphosate exposure was dose-dependently associated with cardiovascular toxicity in animal species [18,39]. Nevertheless, the mechanism underlying the deleterious effect of glyphosate on CVD remain unclear. 

ALP is mainly derived from the liver, bones and intestinal tract, and is an important indicator of hepatobiliary and bone diseases [40,41]. In addition, ALP was reported to play a vital role in the development of CVD via the induction of vascular calcification, endothelial dyshomeostasis and pro-inflammatory activities [42]. In a retrospective analysis of Chinese populations, the author found that serum ALP concentration was positively correlated with arterial stiffness and CVD risk [43]. Serum ALP concentration was identified as a promising predictor of cardiac valve calcification in maintenance hemodialysis patients [44]. Data from the NHANES (1999–2018) also revealed that ALP was positively and significantly correlated with all-cause mortality and CVD mortality in diabetes patients [45]. In line with previous studies, we found that serum ALP was simultaneously linked to increased all-cause mortality and CVD mortality. Considering that glyphosate was markedly and positively associated with serum ALP levels in this study, it is plausible that ALP is involved in the progression of glyphosate-related all-cause mortality and CVD mortality. To validate this hypothesis, we also performed mediation analysis for CVD outcomes, and the results showed that the relationship between glyphosate exposure and all-cause mortality and CVD mortality were significantly mediated by ALP. In addition, the mediating effects of ALP on these associations were incomplete, as the corresponding proportion was 12.1% and 14.0%, respectively. Other contributors or mechanisms might exist, and future studies are required to elucidate the potential mechanism by which glyphosate induces cardiovascular toxicity.

A major strength of this study was that we provided a comprehensive account of the relationship of glyphosate with all-cause mortality and CVD related mortality in the general population with a comparatively large sample size. Moreover, this study constituted the first investigation of the mediating role of ALP in the relationship between glyphosate and CVD related outcomes, providing new insights into the potential mechanism by which glyphosate induced cardiovascular toxicity. The standardized design and strict implementation of NHANES made our findings more credible. However, this study is also limited in some respects. First, the metabolites of glyphosate, such as AMPA and PMIDA, were not measured in the NHANES database. In addition, the exposure to glyphosate was detected only once in the urine samples, which can only reflect actual glyphosate exposure within hours and may lead to bias due to the short half-life of glyphosate [46]. Nonetheless, considering the fact that subjects tend to maintain a stable lifestyle and consistent habits of consuming glyphosate-containing food and materials in their daily life, the single spot urine specimens may also represent the long-term exposure level of glyphosate. Moreover, although we included many covariates in the regression model, some unnoticed confounders may have played a role. Finally, as individuals might be exposed to other environmental pollutants that have simultaneous cardiovascular-toxic potential, our findings might be biased due to co-exposure to other contaminants.

## 5. Conclusions

Our results demonstrated that urinary glyphosate concentration was consistently and dose-dependently associated with increased all-cause mortality and CVD mortality among the general population of US adults. The glyphosate-related increase in serum ALP levels partly mediated the relationships between glyphosate and all-cause mortality and CVD mortality. These findings collectively provide new epidemiological evidence for glyphosate-related cardiovascular illness and highlight the critical role of ALP in the progression of glyphosate-related CVD. More prospective research with a longer follow-up time including more co-exposed toxicants is encouraged to corroborate our results.

## Figures and Tables

**Table 1:** Baseline characteristics of selected individuals in NHANES 2013–2018.

Characteristics	Overall (N = 4031)	Females (N = 2048)	Males (N = 1983)	*p*
Age (years), Mean (SD)	49.2 (17.5)	48.9 (17.3)	49.4 (17.6)	0.360
PIR	2.554 (1.634)	2.516 (1.621)	2.593 (1.646)	0.154
Glyphosate (ng/mL), Mean (SD)	0.498 (0.573)	0.468 (0.542)	0.529 (0.602)	0.001
Creatinine (mg/dL), Mean (SD)	123.48 (79.74)	106.10 (72.00)	141.43 (83.30)	<0.001
Race, frequency (percentage)				0.238
Non-Hispanic Black	806 (20.0%)	420 (20.5%)	386 (19.5%)	
Non-Hispanic White	1613 (40.0%)	799 (39.0%)	814 (41.0%)	
Other Hispanic	424 (10.5%)	232 (11.3%)	192 (9.7%)	
Other Race	1188 (29.5%)	597 (29.1%)	591 (29.8%)	
Education, frequency (percentage)				0.001
Less than high school	800 (19.9%)	367 (18.0%)	433 (21.9%)	
High school or equivalent	901 (22.4%)	440 (21.5%)	461 (23.3%)	
Some college or more	2320 (57.8%)	1236 (60.5%)	1084 (54.8%)	
BMI, frequency (percentage)				<0.001
<25 (kg/m^2^)	1118 (27.9%)	597 (29.3%)	521 (26.5%)	
≥25 (kg/m^2^)–<30 (kg/m^2^)	1261 (31.5%)	552 (27.1%)	709 (36.0%)	
≥30 (kg/m^2^)	1627 (40.6%)	890 (43.6%)	737 (37.5%)	
Alcohol exposure, frequency (percentage)				<0.001
Heavy	418 (10.9%)	114 (5.9%)	304 (16.0%)	
Moderate	1607 (42.1%)	762 (39.6%)	845 (44.6%)	
No	1794 (47.0%)	1048 (54.5%)	746 (39.4%)	
Nicotine exposure, frequency (percentage)				<0.001
≥LLOD	2627 (67.0%)	1254 (62.8%)	1373 (71.4%)	
<LLOD	1292 (33.0%)	743 (37.2%)	549 (28.6%)	
Physical activity, frequency (percentage)				<0.001
No	2262 (56.1%)	1279 (62.5%)	983 (49.6%)	
Moderate	855 (21.2%)	479 (23.4%)	376 (19.0%)	
Vigorous	914 (22.7%)	290 (14.2%)	624 (31.5%)	
Hypertension, frequency (percentage)				0.099
Yes	1723 (42.7%)	849 (41.5%)	874 (44.1%)	
No	2308 (57.3%)	1199 (58.5%)	1109 (55.9%)	
Mortality, frequency (percentage)				0.005
Assumed alive	3807 (94.4%)	1955 (95.5%)	1852 (93.4%)	
Assumed death	224 (5.6%)	93 (4.5%)	131 (6.6%)	
Cerebrovascular cause of death	62 (1.5%)	16 (0.8%)	46 (2.3%)	

Abbreviations: PIR, family poverty income ratio; BMI, body mass index; LLOD, lower limit of detection.

**Table 2:** Association of glyphosate with all-cause mortality and CVD mortality in the general population of adults in the United States, NHANES 2013–2018.

	Weighted Cox Regression with Missing Values of Covariates Imputed		Unweighted Cox Regression with Missing Values of Covariates Imputed		Weighted Cox Regression with Missing Value Deleted		Unweighted Cox Regression with Missing Value Deleted	
	HR (95%CI)	*p*	HR (95%CI)	*p*	HR (95%CI)	*p*	HR (95%CI)	*p*
All-cause mortality								
Model 1	1.77 (1.46 to 2.16)	<0.001	1.67 (1.41 to 1.98)	<0.001	1.86 (1.54 to 2.24)	<0.001	1.71 (1.42 to 2.07)	<0.001
Model 2	1.61 (1.31 to 1.97)	<0.001	1.52 (1.28 to 1.80)	0.012	1.67 (1.37 to 2.03)	<0.001	1.55 (1.28 to 1.88)	<0.001
Model 3	1.29 (1.05 to 1.59)	0.017	1.15 (1.02 to 1.32)	0.036	1.35 (1.08 to 1.69)	0.008	1.17 (1.01 to 1.37)	0.045
CVD mortality								
Model 1	1.84 (1.42 to 2.38)	<0.001	1.67 (1.23 to 2.27)	0.001	1.80 (1.38 to 2.35)	<0.001	1.70 (1.25 to 2.32)	0.001
Model 2	1.67 (1.28 to 2.19)	<0.001	1.51 (1.11 to 2.06)	0.009	1.60 (1.19 to 2.15)	0.002	1.53 (1.12 to 2.09)	0.007
Model 3	1.32 (1.02 to 1.70)	0.042	1.12 (0.82 to 1.52)	0.460	1.24 (0.87 to 1.78)	0.234	1.11 (0.81 to 1.53)	0.498

Model 1 was a crude model; model 2 was adjusted for lifestyle factors (including smoking status, drinking status and physical activity), hypertension and BMI; and model 3 was further adjusted for basic sociodemographic characteristics including age, sex, ethnicity and PIR based on model 2. Abbreviations: CVD, cardiovascular diseases; PIR, family poverty income ratio; BMI, body mass index.

## Data Availability

The datasets analyzed during the current study are available in the NHANES database (https://www.cdc.gov/nchs/nhanes (accessed on 10 June 2024)).
